# Inhibition of miR-331-3p and miR-9-5p ameliorates Alzheimer's disease by enhancing autophagy

**DOI:** 10.7150/thno.47408

**Published:** 2021-01-01

**Authors:** Meng-Lu Chen, Chun-Gu Hong, Tao Yue, Hong-Ming Li, Ran Duan, Wen-Bao Hu, Jia Cao, Zhen-Xing Wang, Chun-Yuan Chen, Xiong-Ke Hu, Ben Wu, Hao-Ming Liu, Yi-Juan Tan, Jiang-Hua Liu, Zhong-Wei Luo, Yan Zhang, Shan-Shan Rao, Ming-Jie Luo, Hao Yin, Yi-Yi Wang, Kun Xia, Si-Yuan Tang, Hui Xie, Zheng-Zhao Liu

**Affiliations:** 1 Department of Orthopedics, Xiangya Hospital, Central South University, Changsha, Hunan 410008, China.; 2 Movement System Injury and Repair Research Center, Xiangya Hospital, Central South University, Changsha, Hunan 410008, China.; 3 Department of Sports Medicine, Xiangya Hospital, Central South University, Changsha, Hunan 410008, China.; 4 Hunan Key Laboratory of Organ Injury, Aging and Regenerative Medicine, Changsha, Hunan 410008, China.; 5 Hunan Key Laboratory of Bone Joint Degeneration and Injury, Changsha, Hunan 410008, China.; 6 Xiangya Nursing School, Central South University, Changsha, Hunan 410013, China.; 7 Institue of Molecular Precision Medicine, Xiangya Hospital, Central South University, Changsha, Hunan 410008, China.; 8 National Clinical Research Center for Geriatric Disorders, Xiangya Hospital, Central South University, Changsha, Hunan 410008, China.; 9 Shenzhen Second People's Hospital, First Affiliated Hospital of Shenzhen University, Shenzhen, Guangdong 518035, China.

**Keywords:** Aβ plaques, Alzheimer's Disease, autophagy, microglia, microRNA

## Abstract

Alzheimer's disease (AD) is currently ranked as the third leading cause of death for eldly people, just behind heart disease and cancer. Autophagy is declined with aging. Our study determined the biphasic changes of miR-331-3p and miR-9-5p associated with AD progression in APPswe/PS1dE9 mouse model and demonstrated inhibiting miR-331-3p and miR-9-5p treatment prevented AD progression by promoting the autophagic clearance of amyloid beta (Aβ).

**Methods:** The biphasic changes of microRNAs were obtained from RNA-seq data and verified by qRT-PCR in early-stage (6 months) and late-stage (12 months) APPswe/PS1dE9 mice (hereinafter referred to as AD mice). The AD progression was determined by analyzing Aβ levels, neuron numbers (MAP2^+^) and activated microglia (CD68^+^IBA1^+^) in brain tissues using immunohistological and immunofluorescent staining. MRNA and protein levels of autophagic-associated genes (*Becn1, Sqstm1, LC3b*) were tested to determine the autophagic activity. Morris water maze and object location test were employed to evaluate the memory and learning after antagomirs treatments in AD mice and the Aβ in the brain tissues were determined.

**Results:** MiR-331-3p and miR-9-5p are down-regulated in early-stage of AD mice, whereas up-regulated in late-stage of AD mice. We demonstrated that miR-331-3p and miR-9-5p target autophagy receptors Sequestosome 1 (*Sqstm1*) and Optineurin (*Optn*), respectively. Overexpression of miR-331-3p and miR-9-5p in SH-SY5Y cell line impaired autophagic activity and promoted amyloid plaques formation. Moreover, AD mice had enhanced Aβ clearance, improved cognition and mobility when treated with miR-331-3p and miR-9-5p antagomirs at late-stage.

**Conclusion:** Our study suggests that using miR-331-3p and miR-9-5p, along with autophagic activity and amyloid plaques may distinguish early versus late stage of AD for more accurate and timely diagnosis. Additionally, we further provide a possible new therapeutic strategy for AD patients by inhibiting miR-331-3p and miR-9-5p and enhancing autophagy.

## Introduction

AD is an irreversible, progressive brain disorder characterized by memory loss and cognitive decline 1, 2. Dementia of patients ranges from the mildest stage to the most server stage, when the person depends completely on others for basic activities of daily living. Research indicates that changes in the brain occur decades before cognitive problems appear 3. However, it is not yet clear what causes these changes. To know the underlying mechanism regulating the AD progression is the first step to achieve the goal of AD treatment, preventing progressive cognitive decline. There remains no effective drug to cure AD 4. Thus, it is vital to develop effective diagnostic tools and new therapeutic strategies from mechanistic studies.

Extracellular Aβ deposition, intracellular accumulation of hyperphosphorylated tau and neurofibrillary tangles formation remain the primary neuropathologic criteria for AD diagnosis 5, 6. The diagnosis and grading of AD are based on some memory and physical assessments evaluating persons who have been suffered from memory loss and cognitive decline 7. AD can be only definitely diagnosed after death, by linking clinical symptoms with an post-mortem examination of Aβ deposition in brain tissue 8. To diagnose people with mild cognitive impairment who may be at great risk for AD remains a challenge due to lack of measurements at early stage. Thus, exploring biomarkers at early stage of AD process is imperative 9, 10. Biomarkers such as microRNAs were demonstrated to play a critical role in AD process 11-14. Studying the role of microRNAs in AD process was of great significance for preventing AD progression.

Aβ was accumulated in the brain of AD patients. Neutralization of Aβ by aducanumab showed a promising perspective for the clinical treatment of AD 15, 16. Autophagy was partially responsible for eliminating the Aβ aggregates and neuronal tau 17-19. The dysfunction of autophagy led to Aβ and tau accumulation and promoted progression of AD 20, 21. However, the regulation of selective autophagy by microRNAs in AD progress remains unknown.

By sequencing the RNAs in nine-month AD and WT mice, two independent groups found that microRNA profile changed during AD process and miR-331-3p was increased in AD mice 22, 23. Examining the altered miRNAs at age 2 months, 4 months, 6 months, 9 months and 12 months by qRT-PCR, Luo* et al.* found that miR-331-3p was increased in 6 months AD mice but decreased in 12 months AD mice as compared with that in WT mice 22. MiR-9 expression was noted to be downregulated in mice primary hippocampal neurons treated with Aβ 24. Lukiw* et al.* showed an upregulation of miR-9 in the temporal cortex and hippocampus of AD patients 25. However, the underlying mechanism is unrevealed that why microRNAs are expressed dynamically and how microRNAs affect AD process.

In this study, we demonstrated the biphasic changes of miR-331-3p and miR-9-5p expression during AD process, which have lower levels at the early-stage and higher levels at the late-stage of AD mice as compared with wild-type (WT) mice. Besides, miR-331-3p and miR-9-5p targeted autophagy receptors *Sqstm1* and *Optn* respectively and affected the selective autophagy pathway during AD process. Our results suggested that autophagy was upregulated in the very beginning of Aβ deposition, which could be defined as the early-stage of AD, and autophagy could be impaired in the late-stage of AD. The autophagic activity was regulated by dynamically expressed microRNAs. Upon inhibiting miR-331-3p and miR-9-5p, AD mice have improved learning and memory.

## Results

### Pathological changes during the AD progression

**Figure 1:**
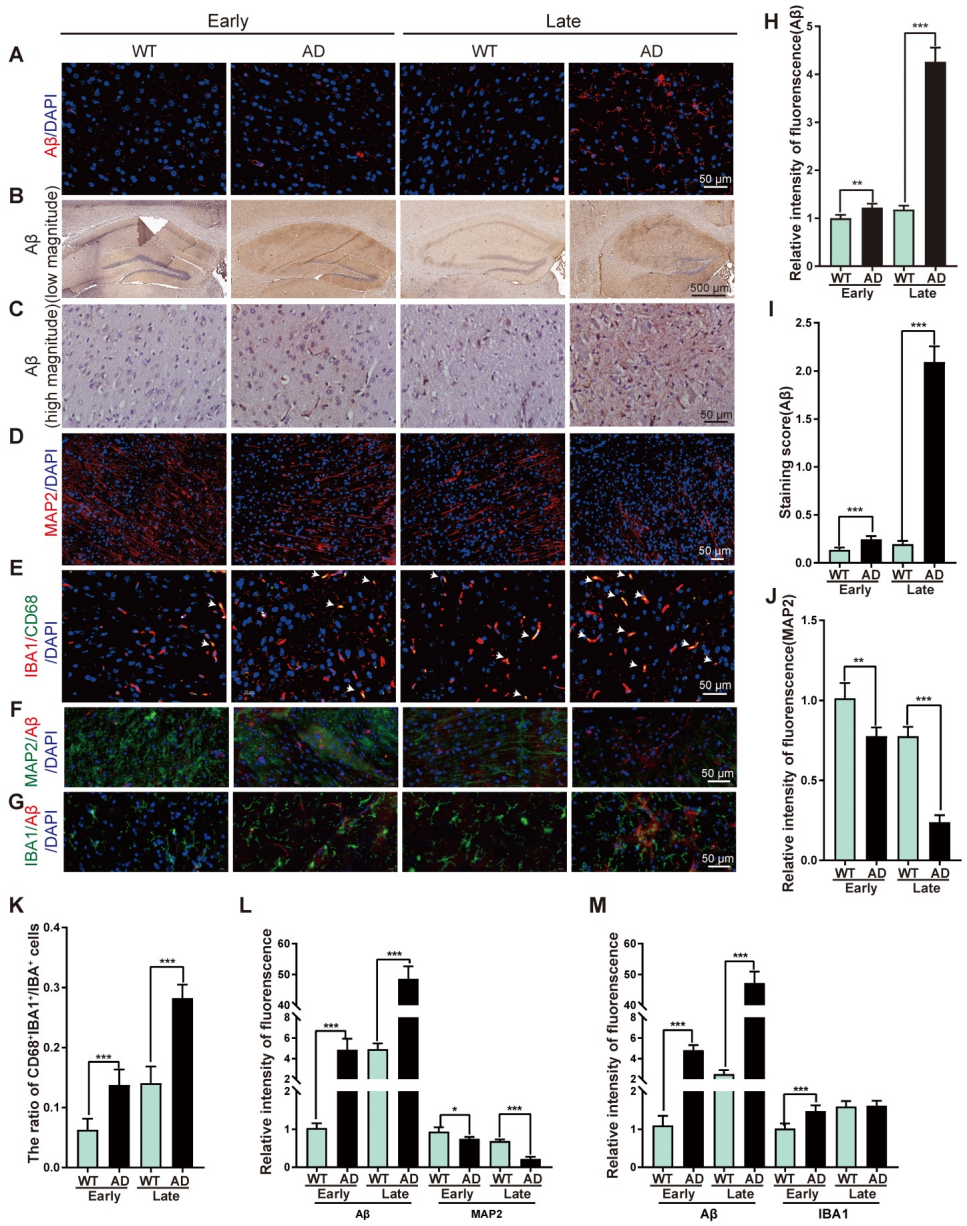
** Representative of the pathological changes at early and late stages of AD. (A)** Immunofluorescent staining of Aβ (red) in brain tissues from WT or AD mice at early-stage or late-stage. Nuclei stained with DAPI were shown in blue. Scale bar, 50 μm.** (B-C)** Representative images of H&E staining of Aβ in hippocampus region of brain tissues from WT or AD mice at early-stage or late-stage at low magnitude (B) and high magnitude (C). **(D)** Immunofluorescent staining of MAP2 (red) in brain tissues from WT or AD mice at early-stage or late-stage. Nuclei stained with DAPI were shown in blue. Scale bar, 50 μm. **(E-G)** Double immunofluorescent staining of IBA1 (red) and CD68 (green) (E), Aβ (red) and MAP2 (green) (F), Aβ (red) and IBA1 (green) (G) in brain tissues from WT or AD mice at early-stage or late-stage. Nuclei stained with DAPI were shown in blue. Arrowheads (E) indicated the CD68 and IBA1 double positive cells. Scale bar, 50 μm. **(H-M)** Quantification of intensity of immunofluorescence of Aβ (H), staining score of Aβ (I), MAP2 (J), the ratio of CD68^+^IBA1^+^:IBA1^+^ cells (K), intensity of immunofluorescence of Aβ and MAP2 (L), Aβ and IBA1 (M) for panel A, C, D, E, F, G, respectively. n = 5 *per* group. For H-M: all data are presented as mean ± sd. ** *P* < 0.01, *** P < 0.001 by one-way ANOVA with Dunettee's post hoc test.

The early and late stages of AD patients and AD mice remain ambiguous in the previous study. For a better description of the presented data, here we designate AD mice ≤ 6-month-old as an early stage of AD mouse model (early-stage of AD), and AD mice ≥ 12-month-old as a late stage of AD mouse model (late-stage of AD). To evaluate the pathology during AD process, Aβ deposition was determined by immunofluorescent staining and showed marginal change in the early-stage, and increased significantly in the late-stage of AD mice as compared with WT mice (**Figure 1A, 1H**). Hematoxylin and eosin (H&E) staining revealed Aβ deposited most in hippocampus of AD mice (**Figure 1B-C, 1I**). Immunofluorescent staining of ionized calcium-binding adapter molecule 1 (IBA1, a microglia marker 26), cluster of differentiation 68 (CD68, highly expressed in activated microglia 27-29), microtubule-associated protein 2 (MAP2, a neuron marker 30) showed more activated microglia cells and less neurons in AD mice than that in WT mice (**Figure 1D-E, 1J-K**). Double immunofluorescent staining revealed higher Aβ accumulation was associated with less neurons and more microglia (**Figure 1F-G, 1L-M, Figure S1**), suggesting microglia activation and neuron damage occurred simultaneously during AD process.

### Biphasic changes of autophagic activity, miR-331-3p and miR-9-5p in AD process

**Figure 2:**
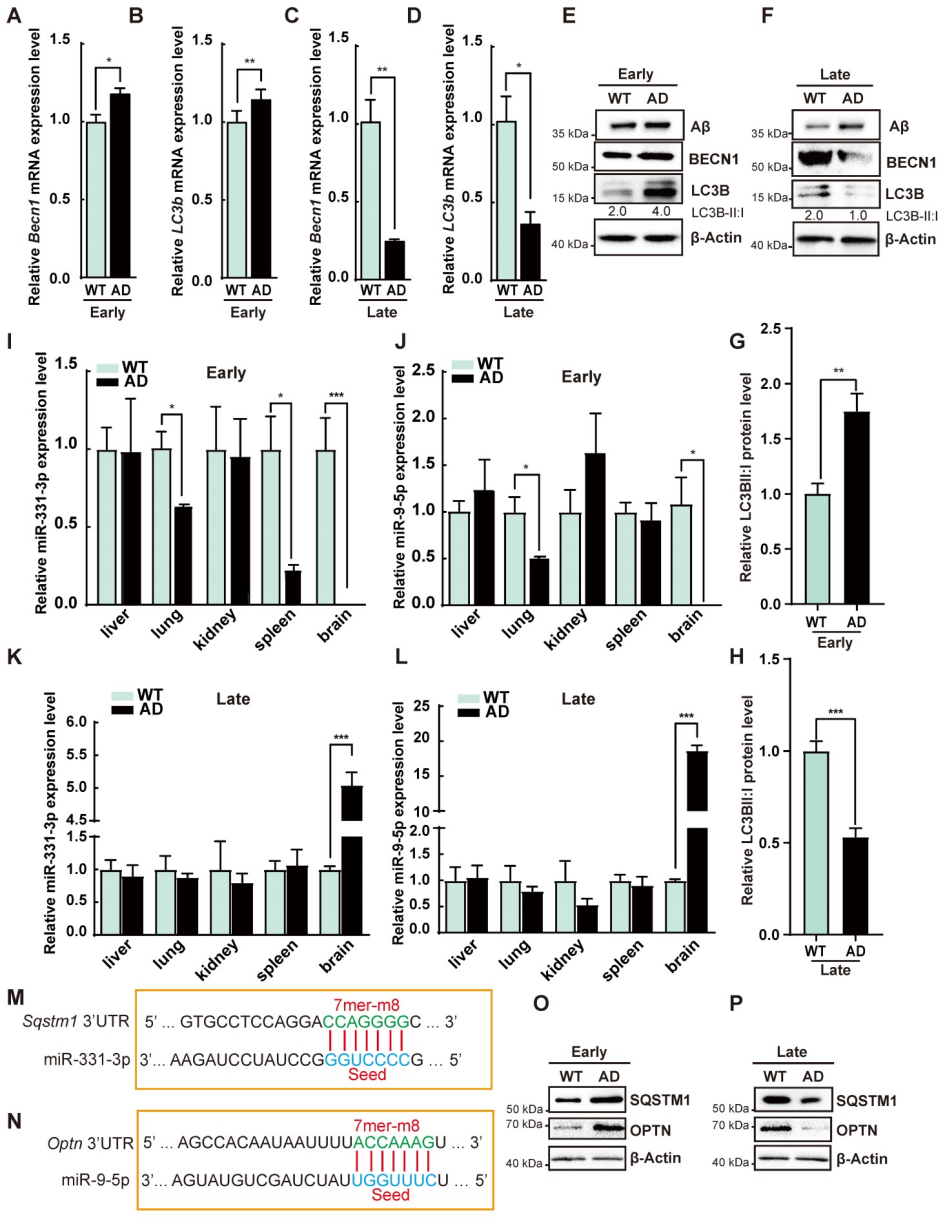
** The autophagic activity was dynamic-changing during the progression of AD. (A-D)** The qRT-PCR analysis of autophagy-associated genes (*Becn1* and* LC3b*) in brain tissues from the WT or AD mice at early-stage or late-stage. n = 3 *per* group. **(E-F)** Western blot analysis of Aβ, BECN1, LC3B of brain tissues from WT or AD mice at early-stage or late-stage. β-Actin was used as a loading control. **(G-H)** Statistical analysis of the relative LC3B-II: I protein level. n = 3 *per* group. **(I-L)** MiR-331-3p and miR-9-5p expression levels in the indicated tissues from WT or AD mice at early-stage or late-stage. n = 3 *per* group.** (M-N)** The predicted miR-331-3p binding site in 3'UTR of* Sqstm1* gene (M) or predicted miR-9-5p binding site in 3'UTR of *Optn* gene (N). Solid lines in the alignments denote the perfect Watson-Crick base-pairing of the seed sequence. **(O-P)** Western blot of SQSTM1 and OPTN in brain tissue from WT or AD mice at early-stage or late-stage. β-Actin was used as a loading control. For A-D, G-L: all data are presented as mean ± sd. * *P* < 0.05, ** *P* < 0.01, *** *P* < 0.001 by unpaired t-test.

To determine autophagic activity in AD process, we analyzed the autophagy-associated gene expression in AD mice. Beclin1 (*Becn1*) and Microtubule-associated proteins 1A/1B light chain 3B (*LC3b*) mRNA levels, protein level of BECN1 and the ratio of LC3B-II:I were increased at early-stage of AD but decreased at late-stage of AD mice as compared with WT (**Figure 2A-H**). On the contrary, AD mice had higher Aβ accumulation at late-stage as determined by western blot (**Figure 2F**). These results suggested enhanced autophagic activity in the early-stage but impaired autophagic activity in the late-stage of AD. To identify miRNA candidates regulating autophagic activity in AD process, miRNA expression profile from the RNA-seq data was analyzed 22, 23. AD mice at 9 months have higher miR-331-3p than WT in two studies 22, 23. MiR-9 is another candidate reported to elevate in the temporal cortex and hippocampus of AD patients 25. The qRT-PCR results from different tissues revealed that miR-331-3p in brain and spleen tissues and miR-9-5p in brain tissue were significantly decreased in early-stage of AD, while both miRNAs in brain tissues manifested higher expression levels in late-stage of AD as compared with WT mice (**Figure 2I-L, Figure S2A-B**). MiR-331-3p and miR-9-5p expression levels in liver, lung, and kidneys were comparable between AD and WT mice (**Figure 2I-L**). Targetscan predicted that autophagy receptors *Sqstm1* and *Optn* were potential targets for miR-331-3p and miR-9-5p respectively (**Figure 2M-N**). AD mice showed higher mRNA and protein levels of SQSTM1 and OPTN at early-stage, and lower mRNA and protein levels of SQSTM1 and OPTN at late-stage of AD mice as compared with WT (**Figure 2O-P, Figure S2C-D**). SQSTM1 and OPTN expression levels were negatively correlated with the miR-331-3p and miR-9-5p expression levels suggested that miR-331-3p and miR-9-5p might play a critical role in AD process by regulating SQSTM1 and OPTN.

### Aβ accumulation was regulated by miR-331-3p and miR-9-5p via targeting *Sqstm1* and *Optn*

**Figure 3:**
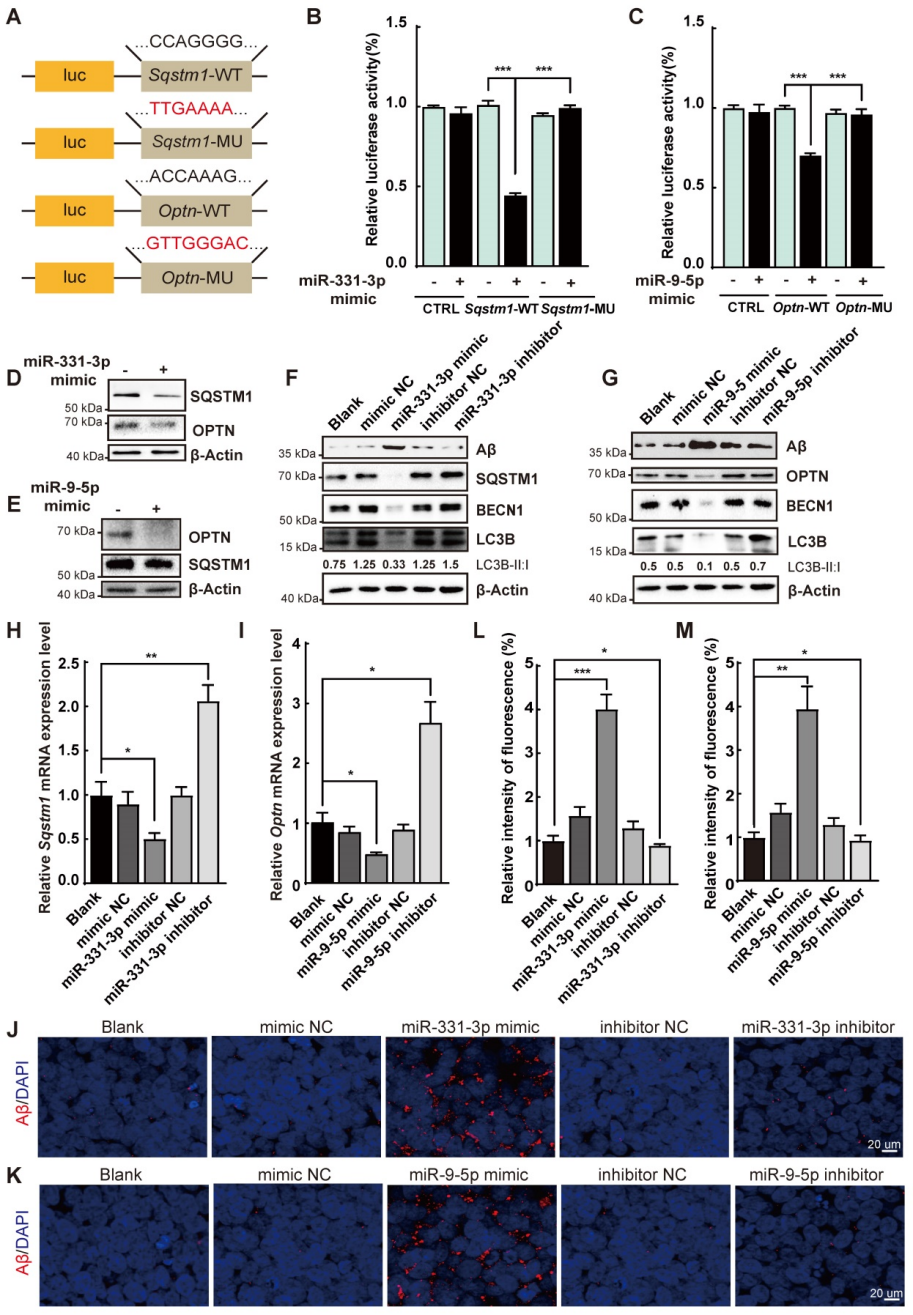
** MiR-331-3p and miR-9-5p impeded Aβ elimination via targeting* Sqstm1* and *Optn.* (A)** Schematic diagram of plasmid construction. The predicted miR-331-3p and miR-9-5p binding sites in 3'UTR of *Sqstm1* and 3'UTR of *Optn* (*Sqstm1*-WT,* Optn*-WT), and target site mutants (*Sqstm1*-MU,* Optn*-MU) were cloned into pmirGLO vector. The seed sequences were shown. **(B)** Luciferase activity was examined in 293T cells co-transfected with indicated plasmids with mimic of miR-331-3p or a mimic control. The luciferase activity was normalized to that of the CTRL vector with a mimic control group. n = 3 *per* group. **(C)** Luciferase activity was examined in 293T cells co-transfected with indicated plasmids with mimic of miR-9-5p or a mimic control. The luciferase activity was normalized to that of the CTRL vector with a mimic control group. n = 3 *per* group. **(D-E)** Western blot of SQSTM1 and OPTN after mimic of miR-331-3p (D) or mimic of miR-9-5p (E) treatment in 293T cells. β-Actin was used as a loading control. **(F-G)** Western blot of Aβ, BECN1, LC3B, SQSTM1 in SH-SY5Y cells transfected with indicated small RNAs. β-Actin was used as a loading control. **(H-I)** The qRT-PCR analysis of *Sqstm1* (F) and* Optn* (G) of SH-SY5Y cells transfected with indicated small RNAs (a mimic control, miR-331-3p mimic or miR-9-5p mimic, an inhibitor control, miR-331-3p inhibitor or miR-9-5p inhibitor). n = 3 *per* group.** (J-K)** Representative images of immunofluorescent staining of Aβ (red) in undifferentiated SH-SY5Y cells treated with indicated small RNAs. Nuclei stained with DAPI were shown in blue. Scale bar = 20 μm. n = 5 *per* group. **(L-M)** Quantification of relative intensity of fluorescence for panel J and K. n = 5 *per* group. For B-C, H-I, L-M: all data are presented as mean ± sd. * *P* < 0.05, ** *P* < 0.01, *** P < 0.001 by one-way ANOVA with Dunettee's post hoc test.

We further scrutinized whether the Aβ deposition was regulated by miR-331-3p and miR-9-5p during the AD process. Luciferase reporter assay was performed to determine whether autophagy receptors* Sqstm1* and* Optn* were the targets of miR-331-3p and miR-9-5p respectively. Fusion expression of 3' untranslated region (UTR) of* Sqstm1* or *Optn* behind the luciferase reporter gene referred as *Sqstm1*-WT, *Optn*-WT; mutated seed sequences of the targeted regions referred as *Sqstm1*-MU, *Optn*-MU (**Figure 3A**). Upon treatment with miR-331-3p or miR-9-5p mimic, luciferase activity in *Sqstm1*-WT or *Optn*-WT was significantly impaired respectively, but marginally changed in *Sqstm1*-MU or *Optn*-MU group (**Figure 3B-C**). SQSTM1 or OPTN protein levels were decreased when 293T cells were treated with miR-331-3p mimic or miR-9-5p mimic respectively as determined by western blot (**Figure 3D-E**). Thus, the results indicated that *Sqstm1* might be the target for miR-331-3p and* Optn* might be the target for miR-9-5p.

To determine whether amyloid plaques was regulated by miR-331-3p and miR-9-5p, SH-SY5Y cells, an *in vitro* cell model for studying function of neuron in AD condition 31, were treated with mimic or inhibitor of miR-331-3p or miR-9-5p respectively. Protein levels of SQSTM1, OPTN, BECN1, and LC3B-II:I ratio were reduced when treated with mimic of miR-331-3p or miR-9-5p, and increased with inhibitor of miR-331-3p or miR-9-5p treatment in SH-S5Y5 cells (**Figure 3F-G**). *Sqstm1* and *Optn* mRNA levels were decreased upon mimic of miR-331-3p or miR-9-5p treatment and increased upon inhibitor of miR-331-3p or miR-9-5p treatment in SH-S5Y5 cells (**Figure 3H-I**), suggesting autophagy-associated genes' expression were inhibited by miR-331-3p or miR-9-5p treatment. Immunofluorescent staining and western blot further revealed higher Aβ accumulation levels in undifferentiated SH-S5Y5 cells with miR-331-3p or miR-9-5p treatment, and lower Aβ accumulation levels when inhibit miR-331-3p or miR-9-5p (**Figure 3J-M**). Furthermore, SH-SY5Y cells were induced to neuronal differentiation with 10 μM retinoid acid (RA) treatment for 7 days 32, 33, and then subjected to mimic or inhibitor of miR-331-3p or miR-9-5p treatment respectively. Consistent with results from undifferentiated SH-S5Y5 cells, RA induced SH-S5Y5 cells showed higher Aβ accumulation levels upon miR-331-3p or miR-9-5p treatment, and lower Aβ accumulation levels when inhibit miR-331-3p or miR-9-5p (**Figure S3A-F).** These results indicated that the elimination of Aβ was regulated by miR-331-3p and miR-9-5p via targeting autophagy receptors *Sqstm1* and *Optn*.

### MiR-331-3p and miR-9-5p antagomirs ameliorated the cognitive decline and aberrant mobility of AD mice at late-stage

In vitro, miR-331-3p and miR-9-5p lead to accumulation of Aβ, we further tested whether inhibiting miR-331-3p or miR-9-5p can ameliorate memory loss and cognitive decline in vivo.

**Figure 4:**
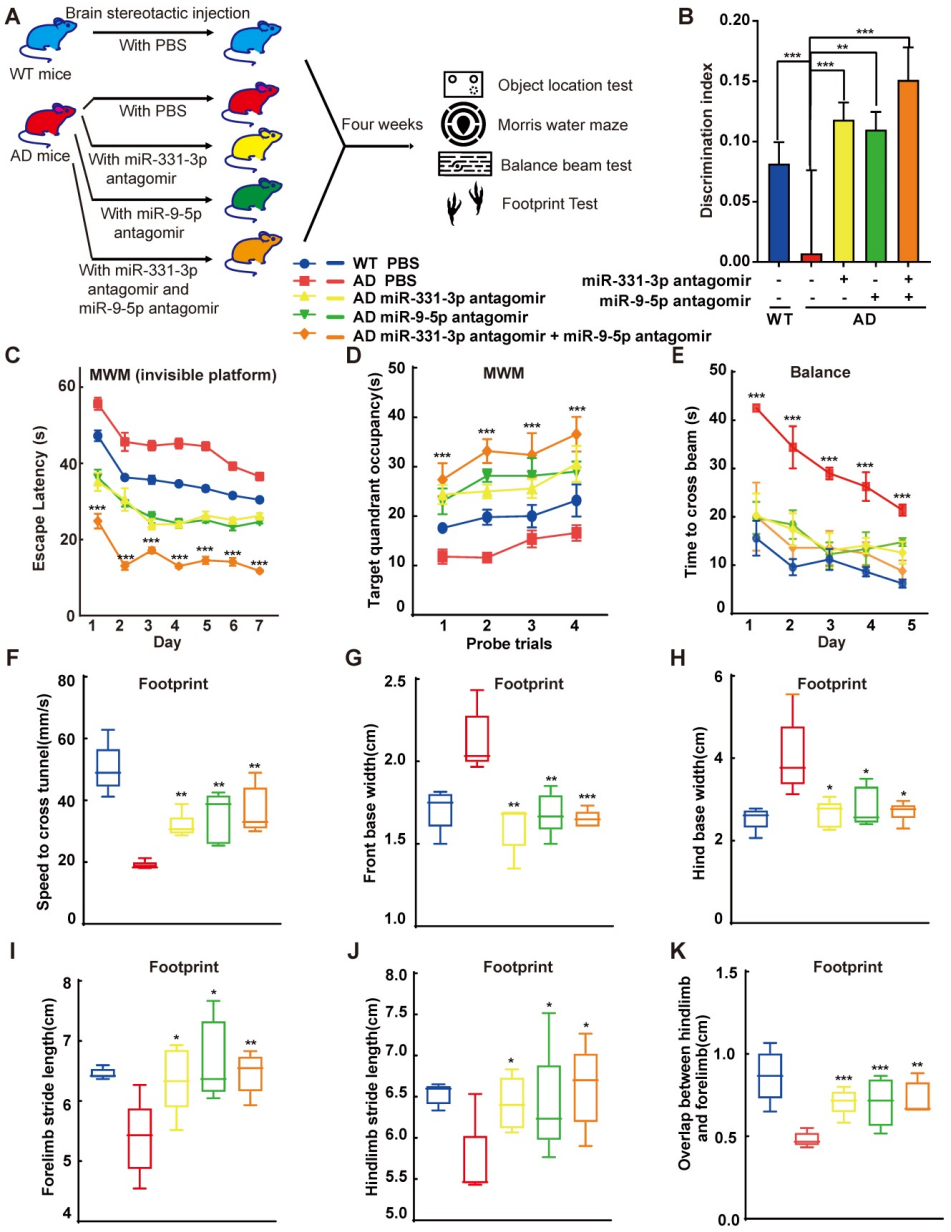
** MiR-331-3p and miR-9-5p antagomirs ameliorated the cognitive decline and aberrant mobility of AD mice. (A)** Scheme of antagomir interventions and subsequent experiments. **(B)** The object location test was performed and discrimination index was determined to analyze the hippocampus-dependent memory. n = 5 *per* group.** (C-D)** The MWM test was performed to analyze the long-term memory. Latency to the invisible platform (C) and target quadrant occupancy (D) were measured. AD mice showed a significant decline in long-term memory performance compared to age-matched wild-type. MiR-331-3p and miR-9-5p antagomirs treatment rescued this effect significantly. n = 5 *per* group. **(E)** The balance beam experiment was carried out for five consecutive days. The latency to cross the beam was evaluated. n = 5 *per* group.** (F-K)** Movement of mice was evaluated by footprint test. Whisker-boxplots that showed various parameters measured in footprint test (speed to cross tunnel, front base width, hind base width, forelimb stride length, hindlimb stride length, overlap between hindlimb and forelimb). n = 5 *per* group. For B-K: all data are presented as mean ± sd. * *P* < 0.05, ** *P* < 0.01, *** P < 0.001 by one-way ANOVAs with Dunettee's post hoc test.

Mice injected with miR-331-3p antagomir or miR-9-5p antagomir, along with the negative control groups were subject to a range of behavioural experiments, which included the object location test (OLT) to determine the hippocampus-dependent spatial learning 34, Morris water maze test (MWM) to check memory improvement, the balance beam test and the footprint test to examine the balance and gaits (**Figure 4A**). To determine the specific brain regions involved in memory decline of AD mice, the OLT which were hippocampus-dependent, were performed. Mice prefer novelty, and they will spend more time investigating the novel object. Mice with hippocampal lesions have impaired spatial contextual learning and consequently demonstrate no preference for novel object 34. After three times of habituation, mice were first tested in a sample stage to explore two identical objects, followed by a test stage substituting one of the familiar objects to a novel location to assess their learning ability (**Figure S4A**). The groups that received one of the antagomirs, or control group spent an almost equal time exploring the objects in the sample stage (**Figure S4B**). In object location test, WT or AD mice treated with miR-331-3p antagomir or miR-9-5p antagomir or both together manifested higher discrimination index than control mice (**Figure 4B, Figure S4C-D**). In the first three days of visual platform training of MWM test, when 12-month-old AD mice were treated with miR-331-3p, miR-9-5p, or both together, the arrival time to the platform reduced compared with littermate controls (**Figure S4E**). Moreover, in the spatial test with invisible platform, AD mice treated with the combination of miR-331-3p and miR-9-5p together showed shorter escape latency than those treated with these two antagomirs respectively (**Figure 4C**). In the part of the target quadrant occupancy, the group that received two antagomirs treatment spent more time in the target quadrant (**Figure 4D**). These observations supported miR-331-3p and miR-9-5p antagomirs could work synergistically to improve memory loss and cognitive decline in AD mice. In the balance beam test, the AD mice treated with miR-331-3p and miR-9-5p antagomirs together also showed shorter time to cross the beam than those treated with miR-331-3p antagomir alone or miR-9-5p antagomir alone, or control group (**Figure 4E**). In footprint test, AD mice treated with miR-331-3p and miR-9-5p antagomirs manifested accelerated speed, longer stride length, shorter base width than those treated with these two antagomirs respectively, or the control mice (**Figure 4F-K**). These results suggested that miR-331-3p and miR-9-5p antagomirs might ameliorate the memory loss and mobility decline in AD mice.

### Aβ elimination was enhanced by miR-331-3p and miR-9-5p antagomirs treatment via activating autophagy

**Figure 5:**
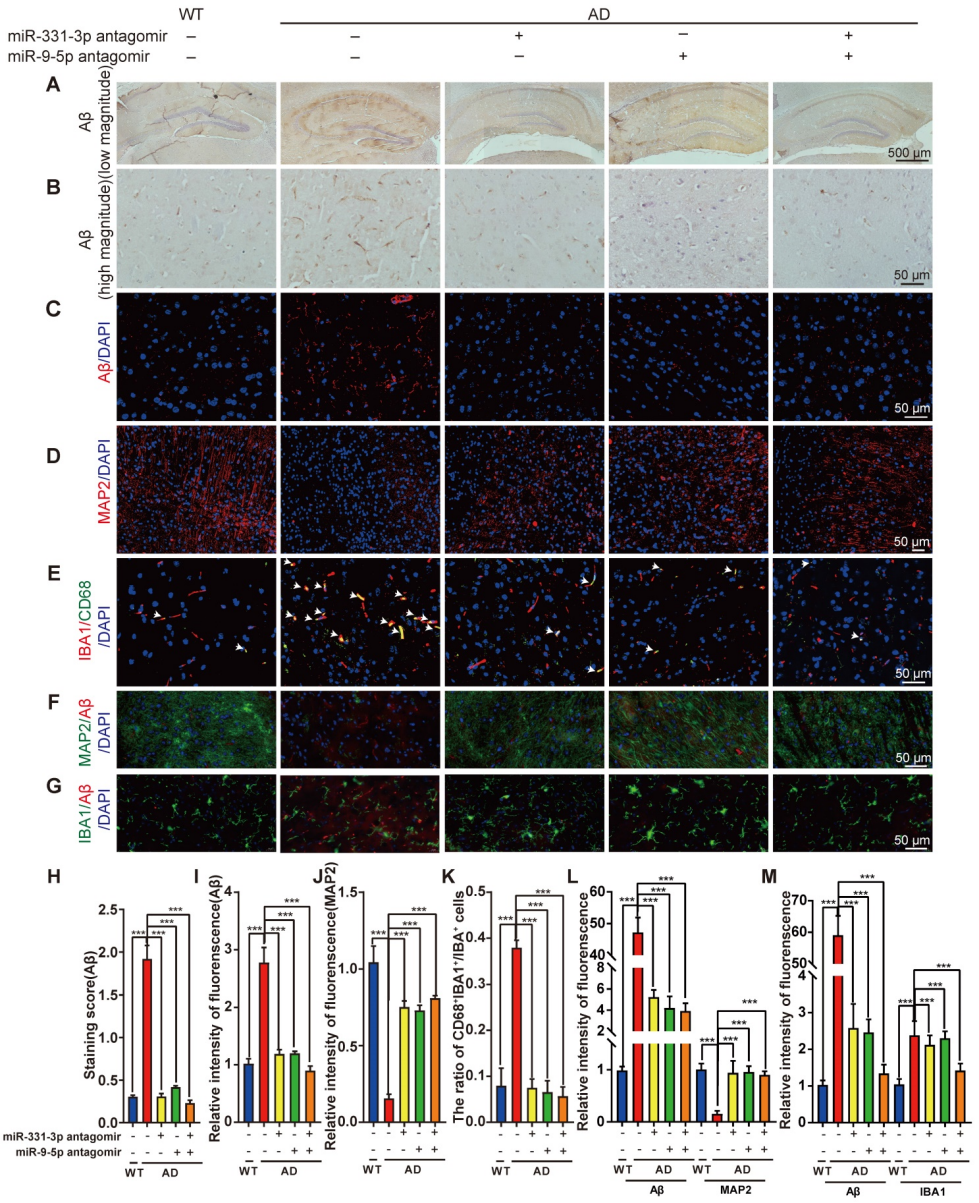
** MiR-331-3p or miR-9-5p antagomirs ameliorate the pathological conditions of AD mice. (A-B)** Representative images of H&E staining of Aβ in brain tissues after stereotactic injection of indicated antagomirs in low magnitude (A) and high magnitude (B). **(C-D)** Immunofluorescent staining of Aβ (red) (C) and MAP2 (red) (D) in brain tissues after stereotactic injection of indicated antagomirs. Nuclei stained with DAPI were shown in blue. Scale bar, 50 μm. **(E-G)** Double immunofluorescent staining of IBA1 (red) and CD68 (green) (E), Aβ (red) and MAP2 (green) (F), Aβ (red) and IBA1 (green) (G) in brain tissues after stereotactic injection of indicated antagomirs. Nuclei stained with DAPI were shown in blue. Arrowheads (E) indicated the CD68 and IBA1 double positive cells. Scale bar, 50 μm. **(H-M)** Quantification of staining score of Aβ (H), intensity of immunofluorescence of Aβ (I), MAP2 (J), the ratio of CD68^+^IBA1^+^:IBA1^+^ cells (K), intensity of immunofluorescence of Aβ and MAP2 (L), Aβ and IBA1 (M) for panel B, C, D, E, F, G, respectively. n = 5 *per* group. For H-M: all data are presented as mean ± sd. *** P < 0.001 by one-way ANOVA with Dunettee's post hoc test.

Furthermore, we scrutinized whether these cognition and mobility improvements of AD mice after miR-331-3p or miR-9-5p antagomir treatment was due to enhanced autophagic activity. The accumulation of Aβ in AD mice treated with miR-331-3p antagomir, or miR-9-5p antagomir, or both together were significantly lower than that in the untreated group (**Figure 5A-C, 5H-I**).

**Figure 6:**
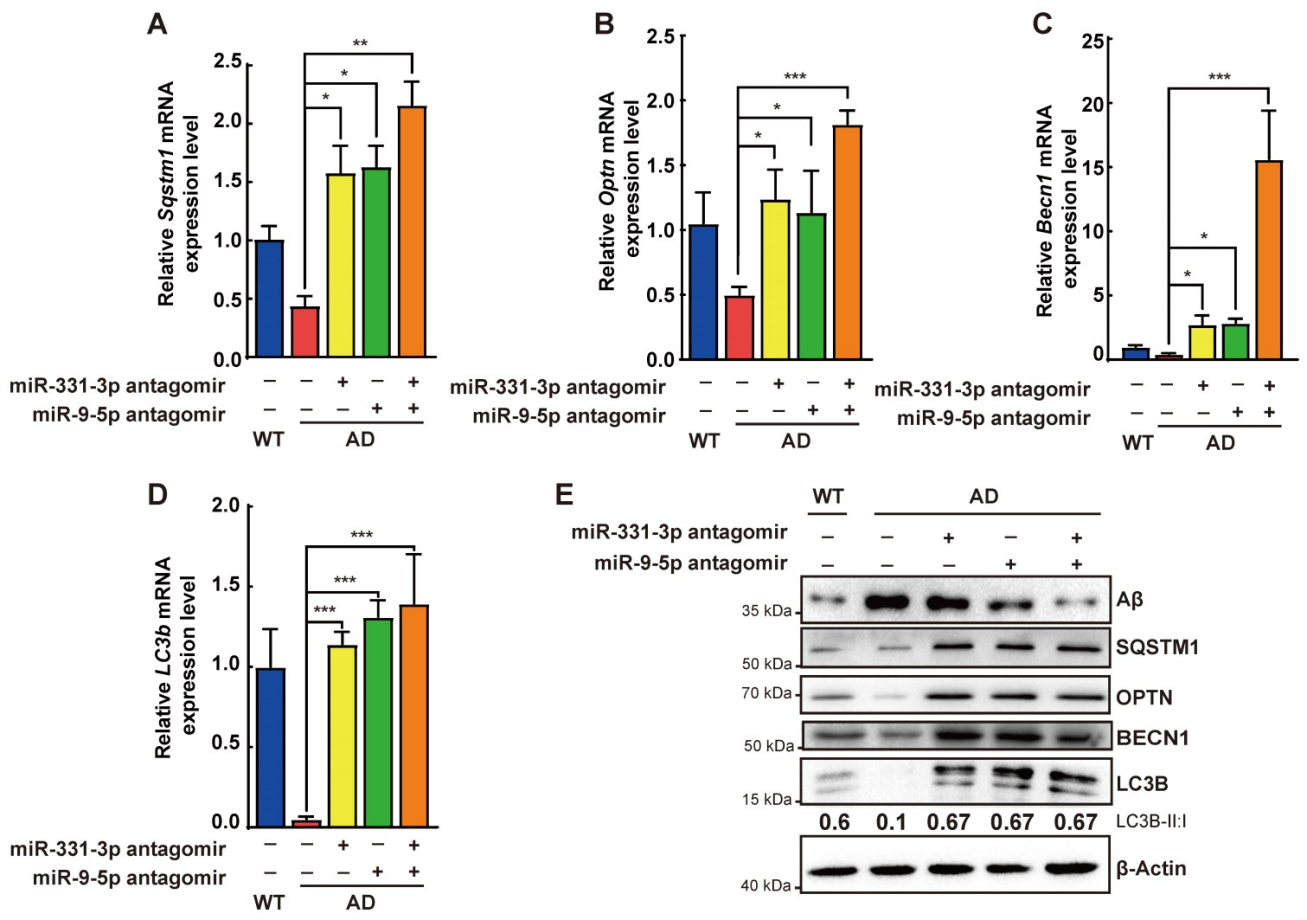
** Aβ elimination was enhanced by miR-331-3p and miR-9-5p antagomirs treatment via activating autophagy. (A-D)** The mRNA expression levels of *Sqstm1* (A), *Optn* (B), *Becn1* (C), *LC3b* (D)in brain tissues after stereotactic injection of indicated antagomirs. n = 3 *per* group. **(E)** Western blots of Aβ, SQSTM1, OPTN, BECN1 and LC3B in brain tissue after stereotactic injection of indicated antagomirs. β-Actin was used as a loading control. For A-D: all data are presented as mean ± sd. * *P* < 0.05, ** *P* < 0.01, *** P < 0.001 by one-way ANOVA with Dunettee's post hoc test.

**Figure 7:**
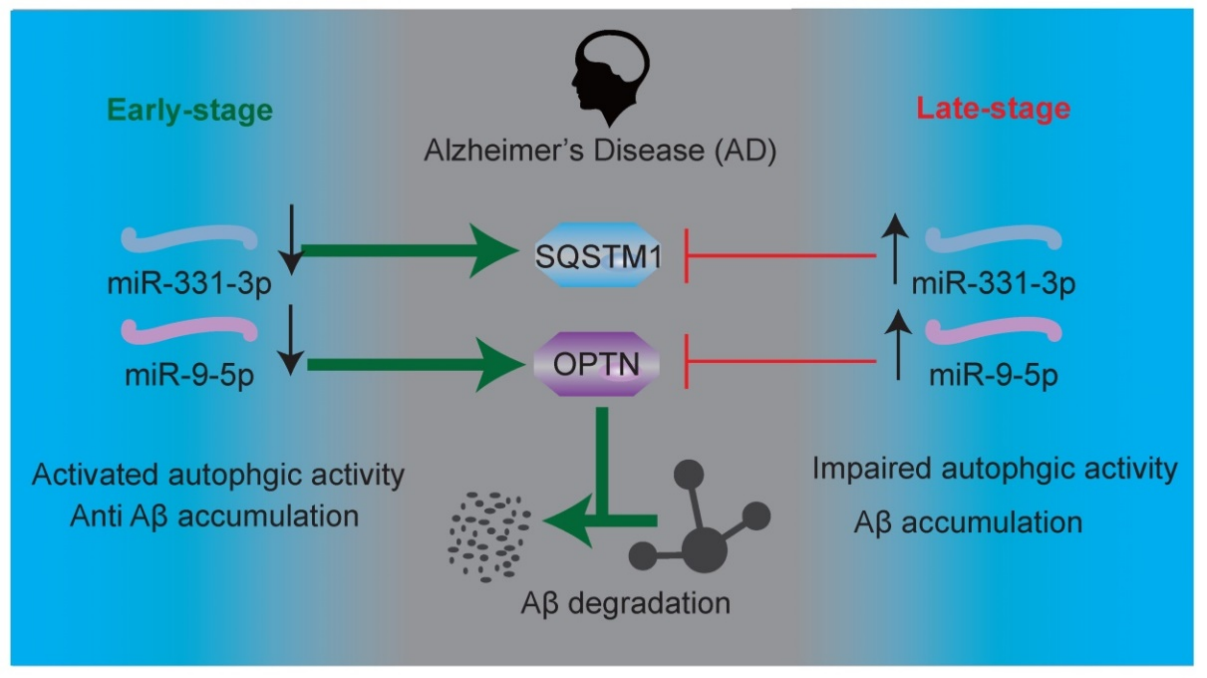
Schematic representation of Aβ elimination regulated by miR-331-3p and miR-9-5p in AD progression.

Compared with the control group, AD mice treated with miR-331-3p antagomir, or miR-9-5p antagomir, or both together showed more neurons, lower number of activated microglia marked by IBA1 and CD68, associated with lower level of Aβ accumulation (**Figure 5D-G, 5J-M, Figure S5**). Moreover, mRNA expression levels of *Sqstm1, Optn, LC3b*, and *Becn1* were higher, protein level of Aβ was lower, protein level of BECN1, SQSTM1, OPTN, ratio of LC3B-II:I were higher in brain tissues of AD mice treated with miR-331-3p antagomir or miR-9-5p antagomir, or both together than that in control AD mice (**Figure 6A-E**). These data implied that miR-331-3p antagomir and miR-9-5p antagomir might activate autophagy and promote Aβ elimination by targeting autophagy receptors in AD mice (**Figure 7**).

## Discussion

Preventing AD progression remains a challenge. Understanding the molecular regulation during AD process is imperative for developing new regime for AD. Herein, our findings demonstrated that biphasic expression of microRNAs during AD process, regulating autophagic activity dynamically. MiR-331-3p and miR-9-5p were downregulated in brain tissues of AD mice at early-stage and upregulated at late-stage, regulating autophagy receptors* Sqstm1* and *Optn* respectively. Targeting miR-331-3p and miR-9-5p could ameliorate the memory loss and mobility decline of AD mice. Assessments of miR-331-3p and miR-9-5p expression levels, along with Aβ accumulation, and autophagic activity may distinguish the early versus late stages of AD progression.

Studies have been reported that microRNAs play a vital role in AD progression. Application of microRNAs used as biomarkers for AD diagnosis was attracted intensive attention 13, 35-37. Biomarker candidates have not been selected due to the high variability between reported data 9. Inconsistencies among studies could be due to several issues including the preparation of samples (samples from blood, cerebrospinal fluid (CSF) or brain tissue), selection of participants (AD patients at different stages) 10, which make the data hard for application. Furthermore, cautions should be taken while using microRNAs as a biomarker of AD, since microRNAs may express dynamically during AD progression 22.

Here, we found that miR-331-3p and miR-9-5p were decreased in the early-stage and increased in late-stage of AD mice. Downregulation of miR-331-3p and miR-9-5p, associated with higher autophagic activity, no significant accumulation of Aβ were observed in early-stage of AD mice, while upregulation of miR-331-3p and miR-9-5p, associated with lower autophagic activity, significant accumulation of Aβ were observed in late-stage of AD mice, suggesting miR-331-3p and miR-9-5p may serve as new biomarkers to distinguish early versus late stages of AD.

Curing AD is the next step after diagnosis. Although donepezil and rivastigmine both contribute to preventing cognition decline in clinical therapy, but still no effective drug curing for AD 7. Autophagy played a significant role in AD pathology 38. Dysfunction of autophagy led to Aβ accumulation 17. But whether interfering autophagy could benefit AD patients remains unclear. In this study, we found miR-331-3p and miR-9-5p targeting autophagy receptors *Sqstm1* and *Optn* respectively. Inhibiting miR-331-3p and miR-9-5p could improve the cognition and mobility of AD mice. We noticed that AD mice treated with both antagomirs were better than the WT mice in MWM test and OLT. These results may be attribute to antagomirs treatment mice had higher SQSTM1 and OPTN expression levels in brain tissues than WT mice (**Figure 6A-B, 6E**), and especially affected hippocampus's function. After all, all the results suggested improvement of cognition and mobility of AD mice when treated with miR-331-3p or miR-9-5p antagomir.

Studies reported previously that activating autophagy could benefit the brain microenvironment supporting the neuronal cell survival 39, 40. Activated microglia is reported to be elevated in AD patients 41, 42. In addition, MiR-9 has been reported to promote microglial activation and neuronal cell death by targeting MCPIP1 43, 44. Consistent with previous studies, AD mice at late-stage had higher miR-9 expression level, increased activated microglia and lower number of neuronal cells in our study. It did not conflict with our finding that miR-9 targeted OPTN and downregulated the autophagic activity. It may be two aspects of miR-9's function. MiR-9 expression level elevates in AD mice at late-stage, activates microglia and leads to neuronal cell death, on the other hand, miR-9 downregulated the autophagic activity by targeting OPTN, destroyed the clearance of Aβ aggregates via autophagy pathway and promote the progression of AD. The underlying mechanism between the miR-9 regulated autophagy pathway associated with microglia activation and neuronal cell death needs more investigations.

## Materials and Methods

### Ethics statement and animal handling

The APPswe/PS1dE9 mice were obtained from the Model Animal Research Center of Nanjing University (Nanjing, China). All procedures for animal uses were in strict accordance with the guidelines of animal welfare set by the World Organization for Animal Health and the Chinese national guideline for animal experiments. All procedures involving animals and their care in this study were approved by the ethical review board at Xiangya hospital of Central South University. Male mice were used for animal experiments.

### Cell culture and cell transfection

Human embryonic kidney (HEK) 293T cells were maintained in Dulbecco's Modified Eagle Medium (DMEM, Gibco) supplemented with 10% FBS, 100 U/mL penicillin, 100 μg/mL streptomycin. SH-SY5Y cells were obtained from Zhong Qiao Xin Zhou Biotechnology Co., Ltd. (ZQ0050, Shanghai, China). SH-SY5Y cells were cultured in Minimum Eagle Medium (MEM, Procell, Wuhan, China) supplemented with 10% FBS, 100 U/mL penicillin, 100 μg/mL streptomycin. Cells were incubated in a humidified incubator at 37 °C with 5% CO_2_. SH-SY5Y cells were treated with 10 μM RA for 7 days to induce neuronal differentiation. For cell transfection, cells were seeded at a density of 20 × 10^4^ per well in a 24-well plate with Opti-MEM. All transfection experiments were performed using Lipofectamine 3000 (Thermo Fisher Scientific, L3000001) following the manufacturer's instruction.

### Brain stereotactic injection

The mice were randomly assigned to the control group, antagomir group. 2.0 µL antagomir solution (50 μM) was injected into each hippocampal region with a small high precision brain stereotaxic instrument (SA-100, Yuyan, Shanghai, China). The injection speed was 0.2 uL/min. After injection, the needle was retained for 2 min for drug absorption, and the needle was withdrawn slowly. Meanwhile, the control group was injected with the same volume of PBS in the hippocampal regions. The mice were then subjected to a series of behavioral experiments after four weeks. The brain samples were harvest with perfusion after behavioral experiments.

### Reagents and plasmids

MiRNA scramble, miR-331-3p mimic, miR-331-3p inhibitor, miR-331-3p antagomir, miR-9-5p mimic, miR-9-5p inhibitor, miR-9-5p antagomir were obtained from RIBOBIO Corporation (Guangzhou, China). To construct luciferase reporter vectors, we cloned the approximately 400bp 3'UTR sequences (extended 200bp on both sides of the microRNA targeting sequences) into pmirGLO between NheI and XbaI. Mutated vectors were constructed into the same site changing the seed sequences of microRNA from G to A, C to T. The sequences were synthesized in GenePharma (Shanghai, China).

### Dural-luciferase assay

293T cells were transfected with pmirGLO (GenePharma, Shanghai, China) with WT or mutated miRNA constructs. Cells were treated with scramble miRNA or miRNA mimics. The activity of luciferase was detected using the Dual-Luciferase® Reporter Assay System (Promega, E1960) according to the manufacturer's instruction. Briefly, cells in 24-well plate were collected after treatment, and lysed with 100 μL passive lysis buffer, we measured the firefly luciferase and Renilla luciferase separately using Varioskan LUX Multimode Microplate Reader (Thermo Fisher Scientific). Relative luciferase activity was calculated as following: Firefly luciferase divided by Renilla luciferase and normalized to the scramble miRNA control group.

### RNA extraction and qRT-PCR analyses

Total RNA was extracted from cells (human 293T cell line, SH-SY5Y cell), and mouse brain tissues using the standard Trizol method (Takara, Beijing, China). For gene expression analysis, synthesis of cDNA was performed using GoScript™ Reverse Transcriptase according to the manufacturer's instruction (Promega, A5001). We validated the RNA-seq data by a self-established stem-loop qRT-PCR. Synthesis of cDNA was performed using mixed reversed transcriptional primers specifically for miR-331-3p (10 μM), miR-9-5p (10 μM), and U6 (10 μM). Amplifications of the microRNAs were carried out by using miR-331-3p-F and URP to amplify miR-331-3p, using miR-9-5p-F and URP to amplify miR-9-5p. U6 was amplified with U6-F and U6-R as normal control. Primers were synthesized in Sangon Biotech (Shanghai, China). Quantitative PCR amplification of indicated genes was performed using GoTaq® qPCR Master Mix (Promega, A6001) on an FTC-3000 real-time PCR machine (funglyn biotech) with GAPDH as a normalization control. After the initial denaturation (2 min at 95 °C), amplification was performed with 40 cycles of 15 s at 95 °C and 60 s at 60 °C. All qPCR data were presented as mean ± sem. The sequences of the primers used for qPCR are listed below: m-GAPDH-F: 5'-AGGTCGGTGTGAACGGATTTG-3', m-GAPDH-R: 5'-TGTAGACCATGTAGTTGAGGTCA-3', m-Sqstm1-F: 5'-GGGAACACAGCAAGCTCATC-3', m-Sqstm1-R: 5'-TGTCAACCTCAATGCCTAGAG-3', m-Optn-F: 5'-ACAGGTGGCTACAGGTATCC-3', m-Optn-R: 5'-TGGGTGTAGGGCAGTTCTTC-3', m-Becn1-F: 5'-CTGGACACTCAGCTCAATGT-3', m-Becn1-R: 5'-ACTATACTCCCGCTGGTACTG-3', m-LC3b-F: 5'-AGCAGCACCCCACCAAGAT-3', m-LC3b-R: 5'-CACCATGCTGTGCCCATTC-3', U6-F: 5'-CTCGCTTCGGCAGCACA-3', U6-R: 5'-AACGCTTCACGAATTTGCGT-3', miR-331-3p-F: 5'-ACACTCCAGCTGGGGCCCCTGGGCCTATC-3', miR-331-3p-R: 5'-CTCAACTGGTGTCGTGGAGTCGGCAATTCAGTTGAGTTCTAGGA-3', miR-9-5p-F: 5'-ACACTCCAGCTGGGACAAAGGAAAACAAGCA-3', miR-9-5p-R: 5'-CTCAACTGGTGTCGTGGAGTCGGCAATTCAGTTGAGTCATACAG-3', URP: 5'-TGGTGTCGTGGAGTCG-3'.

### Immunoblotting analysis

Brain tissues, 293T cells and SH-SY5Y cells extracts were prepared respectively in the RIPA buffer with protease inhibitors [50.0 mM Tris-Cl, 150.0 mM NaCl, 1.0% (v/v) Triton X-100, 1.0% sodium deoxycholate, 0.1% SDS, 2.0 mM sodium pyrophosphate, 25.0 mM β-glycerophosphate, 1.0 mM EDTA, 1.0 mM Na_3_VO_4_, and 0.5 ug/mL leupeptin, pH 7.4] for 30 min and then centrifuged at 14,000 rpm at 4 °C for 15 min to remove the cell debris. Supernatants were separated by SDS-polyacrylamide gel electrophoresis (SDS-PAGE) and blotted onto polyvinylidene fluoride membranes (Millipore, USA). OPTN, SQSTM1, LC3B, BECN1 in the lysates were checked by immunoblotting with Anti-OPTN (Santa Cruz, sc-166576; 1:2000), Anti-LC3 (Sigma, L7543; 1:2000), Anti-SQSTM1 (Abcam, ab56416; 1:3000), Anti-Beclin1 (CST, 3495S; 1:1000), Anti-Aβ (Santa Cruz, sc-28365; 1:1000), Anti-β-actin (Servicebio, GB11001; 1:5000). The immunoreactive bands were visualized by enhanced chemiluminescence reagent (advansta, R-03726-E10) and imaged by the ChemiDoc XRS Plus luminescent image analyzer (Bio-Rad, Hercules, CA, USA).

### Immunohistochemical and Immunofluorescent staining

After antagomir treatment and behavioral experiments, mice were sacrificed for histochemical and immunofluorescence examinations as previously described 45-48. Briefly, animals were deeply anesthetized with 120 μL 0.5% chloral hydrate. After heart perfusion with 30 mL saline, followed by 40 mL 4% paraformaldehyde. Brains were quickly removed and post-fixed in 4% paraformaldehyde for 4-6 h, subsequently cryoprotected in 35% sucrose separately. For Immunohistochemical staining, samples dehydrated by graded ethanol, embedded in paraffin and sliced into 4-μm-thick sections. Aβ accumulation was assayed with anti-Aβ antibody (Santa Cruz, sc-28365; 1:1000). The staining score was calculated using the staining intensity times the cell percentage (-) % × 0 + (+) % × 1 + (++) % × 2 + (+++) % × 3. - indicates negative; + indicates weak staining signal; ++, medium staining signal; +++, strong staining signal. For frozen section examination, samples embedded in optimal cutting temperature (OCT) compound and quick-freeze in liquid nitrogen. After sectioned at 5.0 μm with a Cryostat Microtome, sections incubated at 4 °C overnight with anti-Aβ antibody (Abcam, ab2539; 1:200). After rinsed in PBS for three times (5 min/time), the sections were applied with secondary antibodies goat-anti-rabbit IgG (Cy3) (Abcam; 1:200) at room temperature for 1 h, followed by three rinses (5 min/time) in 100mM PBS, after mounting with Antifade Mounting Medium with DAPI (VECTOR, H-1200), pictures were obtained with the ApoTome.2 Imaging System (ZEISS, Germany). Immunofluorescence assay of MAP2 (Proteintech, 17490-1-AP), IBA1 (Abcam, ab178847), CD68 (Abcam, ab31630) in the brain tissues of WT and AD mice also followed the above steps.

### Morris water maze test

A spatial memory test was performed as previously described with minor modifications 49. The Morris water maze (MWM) is a white circular pool (diameter: 120 cm and height: 50 cm) with a featureless inner surface (XR-XM101, Shanghai Xinruan Information Technology Co. Ltd, Shanghai, China). The circular pool was filled with nontoxic water and kept at 21-23 °C. The pool was divided into four quadrants of equal area. A transparent wooden platform (8 cm in diameter and 20 cm in height) was centered in one of the four quadrants of the pool. There are four prominent visual cues on each side of the four quadrants of the pool. Four habituation training was performed on day 3. The water in the pool was un-dyed, and the platform was visible (1.5 cm above the water surface). Test trials were conducted for seven days (day 4-day 10). The water was white-dyed with non-toxic agents (food-grade titanium dioxide), and the platform was submerged 1.0 cm below the water surface so that it was invisible at the water level. For each daily trial, the mouse was placed into the water maze at one of four randomly determined locations and released allowing the animal to find the hidden platform. After the mouse found and climbed onto the platform, the trial was stopped, and the escape latency was recorded. In order to assess the spatial retention of the location of the hidden platform, a probe trial was conducted 24 h after the last acquisition session. During this trial, the platform was removed from the maze, and each mouse was allowed to search the pool for 60 s before being removed. The time spent in the target quadrant was used as a measure of consolidated spatial memory.

### Balance Beam Test

The ability of the mice to keep balance was evaluated in the balance beam test. Balance beam test was performed as previously described 50. Mice were placed on the same starting point of a horizontal wooden bar (0.9 × 0.9 × 50 cm) 40 cm above the ground and a dark goal box on destination to attract the mouse to run up to this dark and safe environment. The time taken to cross the beam was measured. Mice were given a session and recorded three times per day with a 15-20 min interval and repeated for five successive days.

### Footprint Test

The footprint test was used to describe the mice's gait and performed as previously described 50. The mouse's paws were marked with non-toxic waterproof paint to (red color was employed for forepaws and black for hind paws). Mice were placed on the same starting point of a straight narrow tunnel (10 cm × 10 cm × 70 cm) with a piece of white paper on the bottom to record the footprints. These measurements above were recorded. The parameters included front base width, hind base width, forelimb stride length, hindlimb stride length, the overlap between hindlimb and forelimb and the speed to cross the tunnel.

### The object location test

The object location test (OLT) was performed as previously described 51. The test equipment consists of a square box made of polymethyl methacrylate (40 cm × 40 cm × 40 cm) with external cues (different colour papers in the 4 side walls) to help mice to resolve this spatial memory task. These cues were kept in a constant location throughout the period of testing. Two identical black cylinders (5 cm in diameter, 10 cm tall) are placed at two corners of the device 6 cm from the side wall. Mice release from release corner. During habituation, the mice were allowed to freely explore the instrument for 2 min without objects, once a day for 3 consecutive days. In the Sample Test (T1), two identical subjects were placed, and the mice were allowed to freely explore for 2 min. Then, one object moved to a new location (NL), while the other object remained in familiar location (FL). The Selection Test (T2) was subsequently performed, mice were allowed to explore for 2 min, the exploring time to FL or NL were recorded. Exploration was defined as the mice directed the nose toward the object at a distance of no more than 2 cm. Turning around or sitting on the object was not considered as exploratory behaviour. The times spent by mice in exploring each object during T1 and T2 were recorded. T1: the total exploring time in T1; T2: the total exploring time in T2; % Investigation time of novel location: NL/(NL + FL); Discrimination index: (NL - FL)/(NL + FL).

### Statistical analysis

Data were analyzed using Prism 8 (GraphPad Software). Results were presented as mean ± sd. The statistical significance between various groups or treatments was measured by Student's t test or one-way ANOVA with Dunettee's post hoc test. In all experiments, P-value ≤0.05 was considered to be statistically significant, *** *P* ≤ 0.001, ** *P* ≤ 0.01, * *P* ≤ 0.05, without asterisks means no significance p>0.05.

## Supplementary Material

Supplementary figures.

## Abbreviations

Aβ: amyloid beta
AD: Alzheimer's Disease
Becn1: beclin1
CSF: cerebrospinal fluid
H&E: haematoxylin and eosin
LC3B: microtubule-associated proteins 1A/1B light chain 3B
OPTN: optineurin
OCT: optimal cutting temperature
RA: retinoid acid
Sqstm1/p62: sequestosome 1
UTR: untranslated region
