# Inhibition of miR-331-3p and miR-9-5p ameliorates Alzheimer's disease by enhancing autophagy: Erratum

**DOI:** 10.7150/thno.67227

**Published:** 2021-10-02

**Authors:** Meng-Lu Chen, Chun-Gu Hong, Tao Yue, Hong-Ming Li, Ran Duan, Wen-Bao Hu, Jia Cao, Zhen-Xing Wang, Chun-Yuan Chen, Xiong-Ke Hu, Ben Wu, Hao-Ming Liu, Yi-Juan Tan, Jiang-Hua Liu, Zhong-Wei Luo, Yan Zhang, Shan-Shan Rao, Ming-Jie Luo, Hao Yin, Yi-Yi Wang, Kun Xia, Si-Yuan Tang, Hui Xie, Zheng-Zhao Liu

**Affiliations:** 1 Department of Orthopedics, Xiangya Hospital, Central South University, Changsha, Hunan 410008, China.; 2 Movement System Injury and Repair Research Center, Xiangya Hospital, Central South University, Changsha, Hunan 410008, China.; 3 Department of Sports Medicine, Xiangya Hospital, Central South University, Changsha, Hunan 410008, China.; 4 Hunan Key Laboratory of Organ Injury, Aging and Regenerative Medicine, Changsha, Hunan 410008, China.; 5 Hunan Key Laboratory of Bone Joint Degeneration and Injury, Changsha, Hunan 410008, China.; 6 Xiangya Nursing School, Central South University, Changsha, Hunan 410013, China.; 7 Institue of Molecular Precision Medicine, Xiangya Hospital, Central South University, Changsha, Hunan 410008, China.; 8 National Clinical Research Center for Geriatric Disorders, Xiangya Hospital, Central South University, Changsha, Hunan 410008, China.; 9 Shenzhen Second People's Hospital, First Affiliated Hospital of Shenzhen University, Shenzhen, Guangdong 518035, China.

A black spot on the western blot image of SQSTM1 in Figure 3E was improperly erased by one of the co-authors, and some images in the same group were misplaced in different groups in Figure 3J and Figure 3K. The correction has now been made online. This corrigendum does not affect any results or conclusions of the paper. The authors sincerely apologize for any confusion and inconvenience that it may have caused.

## Figures and Tables

**Figure A:**
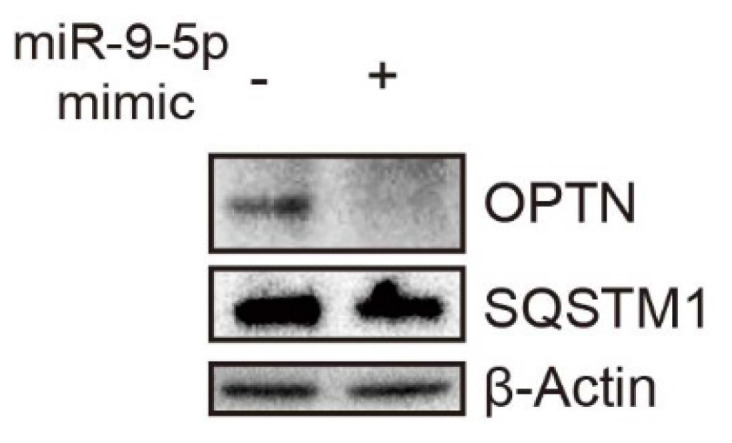
Original Figure 3E. Corrected image

**Figure B:**
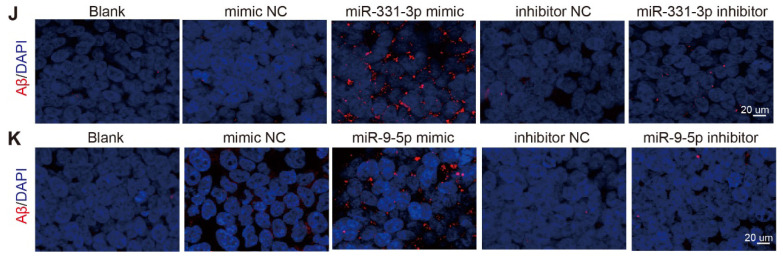
Original Figure 3J-K. Corrected image

