# Supplementary material for: Inhibition of miR-331-3p and miR-9-5p ameliorates Alzheimer's disease by enhancing autophagy
Source: Theranostics. 2021 Jan 1;11(5):2395–409. doi: 10.7150/thno.47408 (PMC7797673; doi:10.7150/thno.47408)
Supplement: Supplementary file 1 — Supplementary figures. [file thnov11p2395s1.pdf]

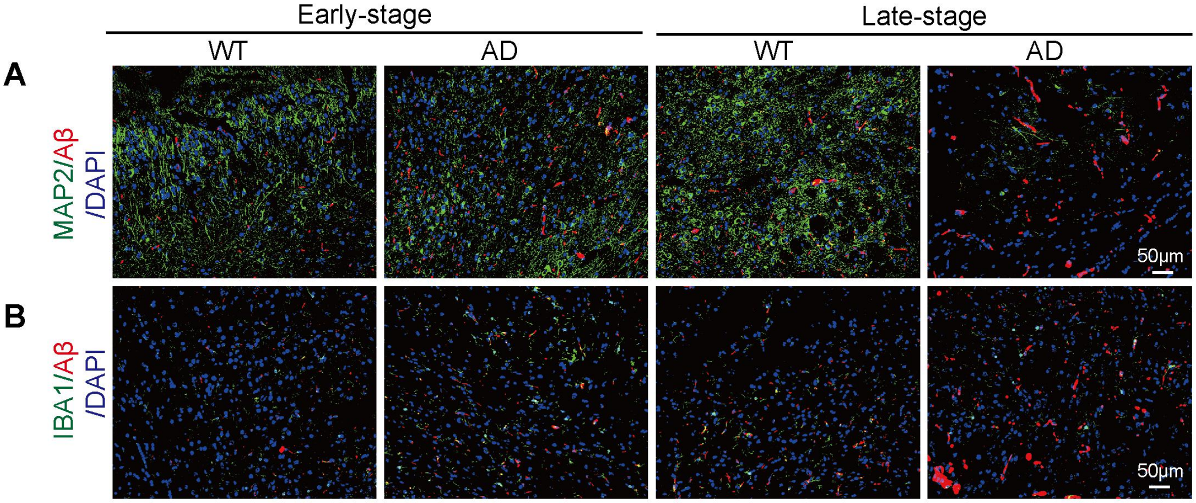

Figure S1. Representative of the pathological changes at early and late stages of AD.

(A-B) Double immunofluorescent staining of Aβ (red) and MAP2 (green) (A), Aβ (red) and IBA1 (green) (B) in brain tissues from WT or AD mice at early-stage or late-stage in high magnitude. Nuclei stained with DAPI were shown in blue. Scale bar, 50 μm.

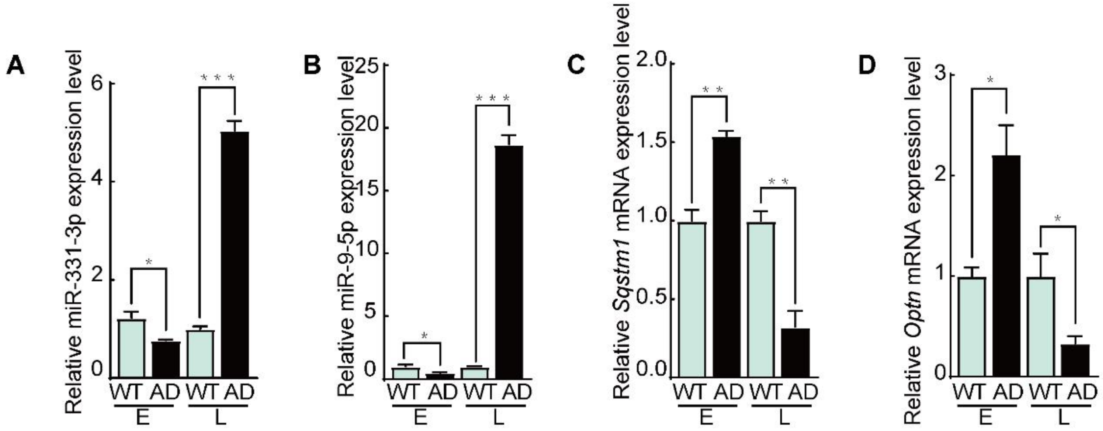

Figure S2. MiR-331-3p and miR-9-5p were dynamic-changing in the brain during the progression of AD.

(A-B) The miR-331-3p and miR-9-5p expression levels in the brain tissues from WT or AD mice at early-stage or late-stage. n = 3 *per* group. **(C-D)** The qRT-PCR analysis of *Sqstm1* and *Optn* in

the brain tissues from WT or AD mice at early-stage or late-stage. n = 3 *per* group. For A-D: all data are presented as mean ± sd. * $P < 0.05$, ** $P < 0.01$, *** $P < 0.001$ by unpaired t-test.

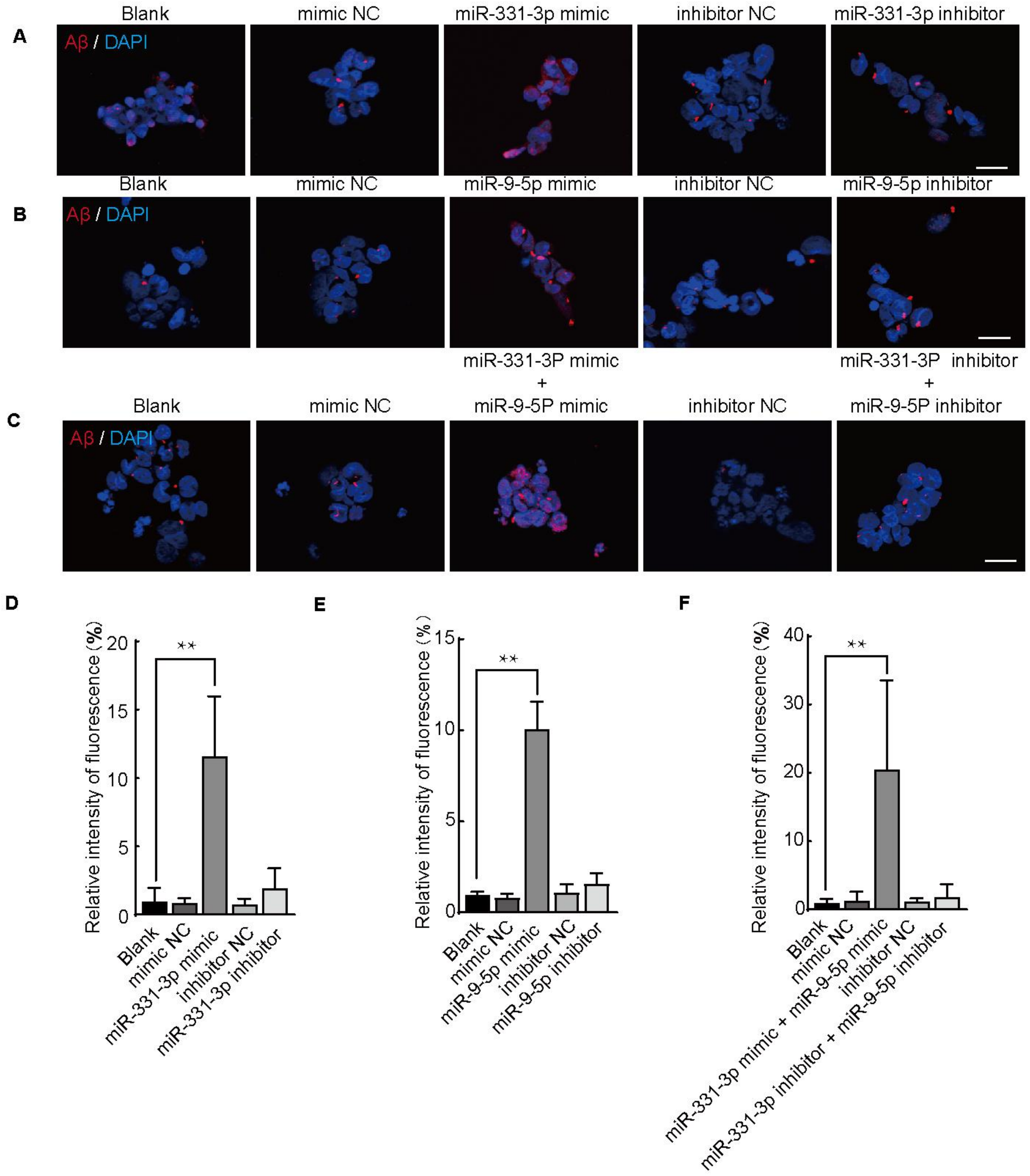

Figure S3. MiR-331-3p and miR-9-5p impeded Aβ elimination in differentiated SH-SY5Y. **(A-C)** Representative images of immunofluorescent staining of Aβ (red) in differentiated SH-SY5Y cells treated with indicated small RNAs. Scale bar = 20 μm. **(D-F)** Quantification of

relative intensity of fluorescence for panel A-C. n = 5 *per* group. For D-F: all data are presented as mean ± sd. ** $P < 0.01$ by one-way ANOVA with Dunettee's post hoc test.

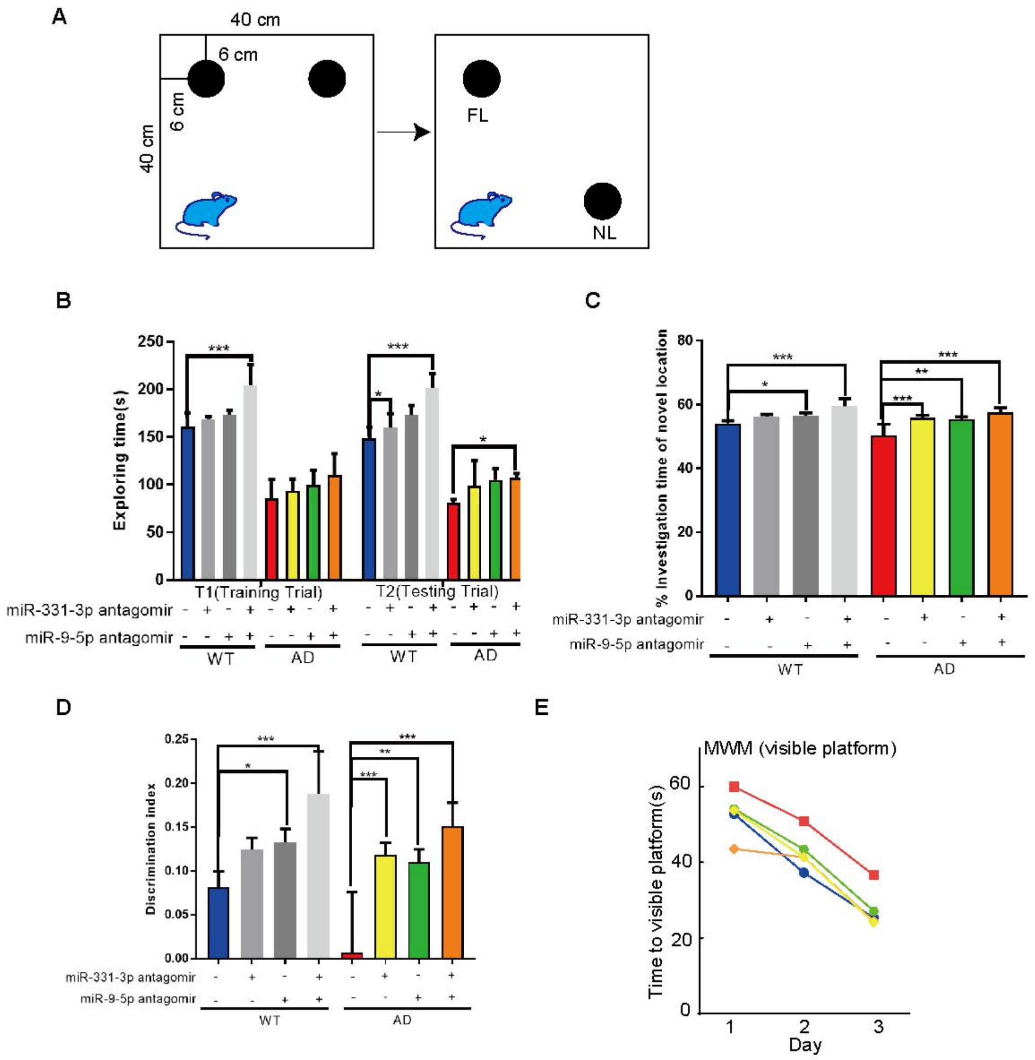

Figure S4. MiR-331-3p and miR-9-5p antagomirs ameliorated the memory and cognitive decline of AD mice.

(A) Schematic representation of object location test. FL, familiar location; NL, new location. **(B-D)** The exploring time (B), % investigation time of novel location (C) and discrimination index (D) were analyzed by object location test. n = 5 *per* group. **(E)** Time to the visible platform

were measured by the MWM test. For B-D: all data are presented as mean ± sd. * $P < 0.05$, ** $P < 0.01$, *** $P < 0.001$ by one-way ANOVA with Dunettee's post hoc test.

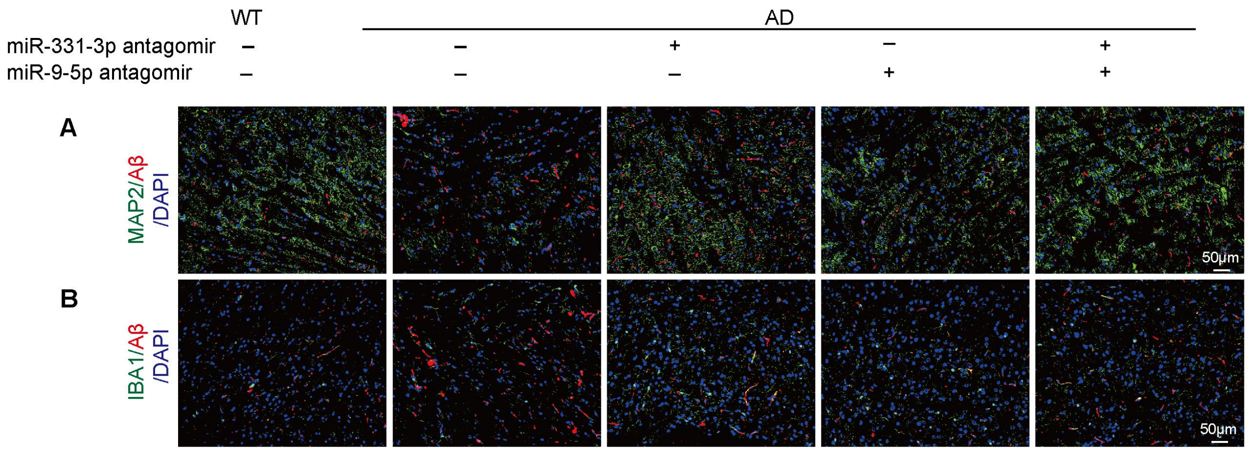

Figure S5. MiR-331-3p or miR-9-5p antagomirs ameliorate the pathological conditions of AD mice.

(A-B) Double immunofluorescent staining of Aβ (red) and MAP2 (green) (A), Aβ (red) and IBA1 (green) (B) in brain tissues after stereotactic injection of indicated antagomirs. Nuclei stained with DAPI were shown in blue. Scale bar, 50 μm.
